# DJAN: Deep Joint Adaptation Network for Wildlife Image Recognition

**DOI:** 10.3390/ani13213333

**Published:** 2023-10-26

**Authors:** Changchun Zhang, Junguo Zhang

**Affiliations:** 1 School of Technology, Beijing Forestry University, Beijing 100083, China; zhangchangchun@bjfu.edu.cn; 2 State Key Laboratory of Efficient Production of Forest Resources, Beijing 100083, China; 3 Key Laboratory of National Forestry and Grassland Administration on Forestry Equipment and Automation, Beijing 100083, China

**Keywords:** transfer learning, wildlife recognition, distribution discrepancy, domain adaptation, deep learning

## Abstract

**Simple Summary:**

Identifying wildlife species is crucial in various wildlife monitoring tasks. In this paper, a wildlife image recognition approach is implemented based on deep learning with a joint adaptation network. This paper presents a joint adversarial learning approach and a cross-domain local and global representation learning approach. Utilizing the two approaches, a Deep Joint Adaptation Network model for wildlife image recognition is designed. The proposed model can yield high accuracy in wildlife image recognition and is beneficial to improve the generalization ability in complex environments. Our research is of the utmost importance for wildlife recognition and wildlife biodiversity monitoring.

**Abstract:**

Wildlife recognition is of utmost importance for monitoring and preserving biodiversity. In recent years, deep-learning-based methods for wildlife image recognition have exhibited remarkable performance on specific datasets and are becoming a mainstream research direction. However, wildlife image recognition tasks face the challenge of weak generalization in open environments. In this paper, a Deep Joint Adaptation Network (DJAN) for wildlife image recognition is proposed to deal with the above issue by taking a transfer learning paradigm into consideration. To alleviate the distribution discrepancy between the known dataset and the target task dataset while enhancing the transferability of the model’s generated features, we introduce a correlation alignment constraint and a strategy of conditional adversarial training, which enhance the capability of individual domain adaptation modules. In addition, a transformer unit is utilized to capture the long-range relationships between the local and global feature representations, which facilitates better understanding of the overall structure and relationships within the image. The proposed approach is evaluated on a wildlife dataset; a series of experimental results testify that the DJAN model yields state-of-the-art results, and, compared to the best results obtained by the baseline methods, the average accuracy of identifying the eleven wildlife species improves by 3.6 percentage points.

## 1. Introduction

**Figure 1:**
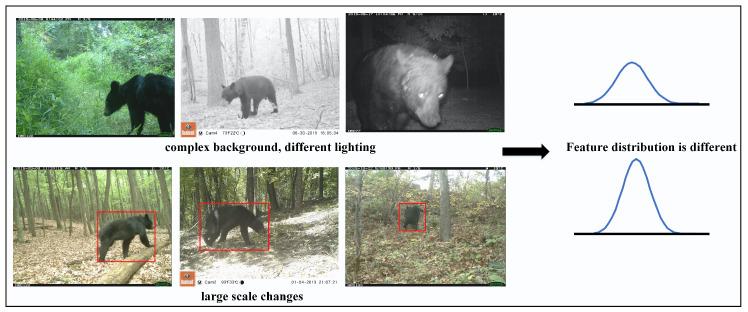
The characteristics of wildlife images.

Wildlife identification is a crucial component of monitoring and conserving biodiversity in the wild. Wildlife monitoring serves as the foundation for wildlife conservation management [1] and plays an importance role in this regard. Through collecting and analyzing data on various aspects of wildlife, such as habitat utilization, migration patterns, quantity distribution, and behavioral activities, monitoring provides valuable information [2] and insights for conservation organizations and government agencies. These data unveil key issues related to species population status, habitat quality, and habitat connectivity, which, in turn, contribute to the development of effective conservation strategies and management plans. Thus, the identification of wildlife species is essential for species diversity detection and the conservation of rare and endangered wildlife. Currently, camera traps are the mainstream method for wildlife species monitoring [3,4,5]. However, manual identification of monitoring images is known to suffer from the challenges of high intensity and low efficiency. With the rapid development of artificial intelligence technology, automatic wildlife identification methods based on deep networks have demonstrated excellent performance on specific datasets [6,7,8,9]. A deep convolutional neural network for automatic identification of wildlife was proposed [10] and achieved a Top-1 accuracy of 88.9% and a Top-5 accuracy of 98.1% on the Serengeti dataset, a public wildlife dataset. Trnovszky et al. [11] achieved a recognition accuracy of 98.0% using an improved LeNet model on a dataset containing five wildlife species. Verma et al. [12] addressed the interference of cluttered scene images, which do not contain individual animals, in wildlife recognition in monitoring datasets. They utilized deep convolutional neural networks (DCNNs) to extract features from cluttered scene images and achieved cluttered scene image recognition based on VGGNet and ResNet, further improving the recognition accuracy of high-value wildlife monitoring images. Schneider et al. [13] focused on the problem of model generalization in unknown scenes and compared the performance of different deep learning methods on various data scenarios. Vargas-Felipe et al. [14] used convolutional neural networks to recognize wildlife in monitoring images, and their recognition accuracy was significantly better than that of traditional recognition methods. Schindler [15] combined instance segmentation networks with action recognition networks to detect wildlife and perform simultaneous action recognition, providing more-diversified ecological analysis data for wildlife monitoring. However, in the real world, as depicted in Figure 1, factors such as different backgrounds, varying lighting conditions, and diverse shooting scales can lead to changes in feature distributions within the same class of images [16,17,18,19]. These factors can result in suboptimal performance of existing deep learning algorithms, posing significant challenges to image recognition.

With the successful application of deep domain adaptation in various fields such as pattern recognition and computer vision, the learning paradigm based on deep transfer learning updates or ‘transfers’ models from one domain to another, breaking the limitations of traditional deep learning models that require a large amount of labeled data as a prerequisite and also overcoming the strict requirement for data to follow the same distribution. Therefore, domain-adaptation-based wildlife image recognition has become a current research hotspot and has yielded good results. Norouzzadeh et al. [20] applied transfer learning to pre-trained convolutional neural network models such as AlexNet, NiN, VGGNet, and GoogLeNet, and compared the performance of the two models in automatically detecting animal species in CT image datasets. A transfer-learning-based wildlife image classification method [21] is proposed that is based on the Xception network, achieving an average accuracy rate of 99.01%, which is a 57.82% improvement in accuracy compared to standard convolutional neural network methods. Thangaraj et al. [22] utilized the concept of transfer learning fine-tuning and fine-tuned mainstream network models such as DenseNet169 and Xception for animal individual recognition. Compared with other models, the InceptionResNetV2 model achieved an accuracy rate of 94.82%. By employing deep domain adaptation approaches, not only are the limitations of traditional deep learning methods requiring a large amount of labeled samples and samples following the same distribution overcome, but also data-driven optimization of recognition models with strong generalization capability can be achieved. However, the aforementioned models are still limited to optimizing DCNN models for wildlife recognition through transfer learning fine-tuning approaches, and they cannot be widely generalized and applied. The diversity of wildlife species, the similarities between classes, and the differences within classes increase the difficulty of wildlife recognition, requiring the establishment of models with strong feature extraction and generalization capabilities for wildlife recognition.

The above-mentioned transfer learning methods only utilize the idea of fine-tuning, which can partially leverage knowledge from the source domain to assist the learning of the target domain and mitigate domain differences. However, in order to further alleviate domain discrepancies and enhance the model’s generalization performance, some researchers have proposed a series of deep transfer learning methods. Sun et al. [23] proposed the Correlation Alignment (CORAL) method, which utilizes a linear transformation to align the second-order statistical information between the source domain sample distribution and the target domain sample distribution, aiming to minimize domain differences. However, CORAL relies on linear transformations and cannot be trained in an end-to-end style. To address such a limitation, Sun et al. [24] further extended the CORAL algorithm and introduced the Deep Correlation Alignment (DCORAL) algorithm. DCORAL embeds CORAL directly into a deep network, constructing a differentiable loss function to minimize cross-domain correlation differences. With the advent of Generative Adversarial Networks (GANs) [25], adversarial domain adaptation methods [26,27,28,29,30] have emerged, which aim to generate domain-transferable features through the adversarial game between a feature generator and a domain classifier. Inspired by the idea of GANs, a domain-adversarial neural network [31] is proposed, which involves three modules: a shared feature extractor cross-domain, a label classifier, and a domain classifier. The feature extractor and label classifier aim to minimize classification errors in the source domain, ensuring that the learned features are discriminative. At the same time, the domain classifier maximizes the domain classification error, encouraging domain-invariant feature distributions. To balance the competition between the feature extractor and label classifier with the domain classifier during training, a gradient reversal layer (GRL) is introduced. GRL works by reversing the gradients of the domain classifier’s loss so that while the domain classifier aims to minimize its loss, the feature extractor maximizes its loss. This is achieved by flipping the sign of the gradients and propagating them back to the feature extractor. The gradient reversal layer enables the network to ensure domain confusion between the domains while preserving the discriminative power of the learned features.

Based on DANN, Long et al. [32] presented a joint domain adaptation network model that outperforms domain-adversarial network models on multiple image classification databases. The JDAN model considers how to match the joint activation distributions of multiple layers in the source and target domains by constraining the Joint Maximum Mean Discrepancy (JMMD). It uses a multi-layer neural network to parameterize JMMD and employs adversarial learning methods to learn discriminative features. Chen et al. [33] discovered that although adversarial domain adaptation methods enhance the transferability of features, they can decrease the discriminability of feature classes. To address this, they introduced batch spectral penalization during training to ensure that the differences between feature values are not too large, thereby preserving feature discriminability. The shared network feature extraction mechanism prevents the generation of domain-specific information for each domain. To overcome this, Tzeng et al. [34] used a weight non-sharing strategy to independently generate features for each domain. Unlike DANN [31], this method allows the feature extractor to generate more domain-specific features due to the non-shared parameters. Volpi et al. [35] utilized a feature generator for data augmentation within the source domain feature space. They employed a domain classifier to distinguish between the generated and authentic features, ultimately aligning the distribution of the augmented data with that of the target domain.

**Figure 2:**
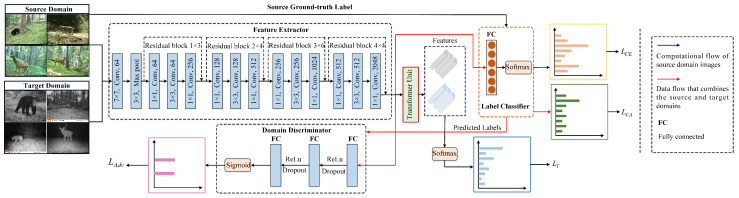
Structure diagram of Deep Joint Adaptation Network. Note: $L_{CE}$ denotes the loss function of optimizing the source domain data; $L_{CA}$ is correlation alignment loss, which is defined as the measurement between the second-order statistics information of label classifier prediction of the training and test images; $L_{Adv}$ indicates the objective optimization function for the final domain discriminator *D* and feature extractor *G* in adversarial domain alignment; $L_{T}$ represents a transformer loss.

The aforementioned methods that use cross-domain feature alignment based on marginal probability distributions overlook the effect of conditional probability distributions in improving network transfer gain. Therefore, researchers have introduced class information to align the conditional probability distributions cross-domain. A multi-adversarial domain adaptation method is proposed [36] to capture the multi-modal structure of data. For a K-class classification problem, this method introduces K domain discriminators, where each domain discriminator matches the samples from the source and target domains with the same label. Specifically, it performs soft classification of the data based on probabilities generated by the class classifier. When the label space of the target domain is a subset of the label space of the source domain, i.e., in the case of partial domain adaptation, it needs to select a subset of source domain samples from the shared label space. There are also some methods that, although they do not employ multiple domain discriminators, achieve similar effects by introducing class information and are therefore classified as multi-adversarial methods. A conditional domain adversarial networks is presented [37], which combines instance weights with adversarial feature learning to capture the multi-modal structure hidden under complex data distributions, leading to effective knowledge transfer. However, during the process of translating technology into practical applications, existing methods have two limitations. Firstly, they rely on single-domain adaptation and class information, neglecting the relationship between adversarial domain adaptation and domain alignment. Secondly, they fail to capture the long-term relationship between local and global feature representations, which would help better understand the overall structure and relationships within images. To address these challenges, a Deep Joint Adaptation Network (DJAN) is proposed in this paper, as shown in Figure 2. Specifically, it incorporates class information into the domain adversarial network to explore more fine-grained transferable features. Additionally, it further exploits more transferable features across domains via considering correlation alignment. It is worth noting that the domain-transferable features learned in this process exhibit strong transferability for different images and different transfer tasks, demonstrating the generalization capability of this method. It effectively improves the accuracy of wildlife image recognition while enhancing the usability and scalability of the approach. In the learning process, a transformer unit is proposed to capture the long-term relationship between local and global feature representations, achieving optimal transfer effects. In summary, this paper presents three main contributions:

- A correlation alignment constraint and the strategy of conditional adversarial training are proposed to enhance the capability of individual domain adaptation modules.
- Combining the correlation alignment constraint and the strategy of conditional adversarial training, a transformer unit is proposed to capture the long-range relationships between the local and global feature representations, which facilitates better understanding of the overall structure and relationships within the image.
- A series of experimental results prove that our approach yields state-of-the-art results, and, compared to the best results achieved by the baseline methods, the average accuracy of identifying the eleven wildlife species improves by 4.7%.

## 2. Materials and Methods

### 2.1. Dataset

**Figure 3:**
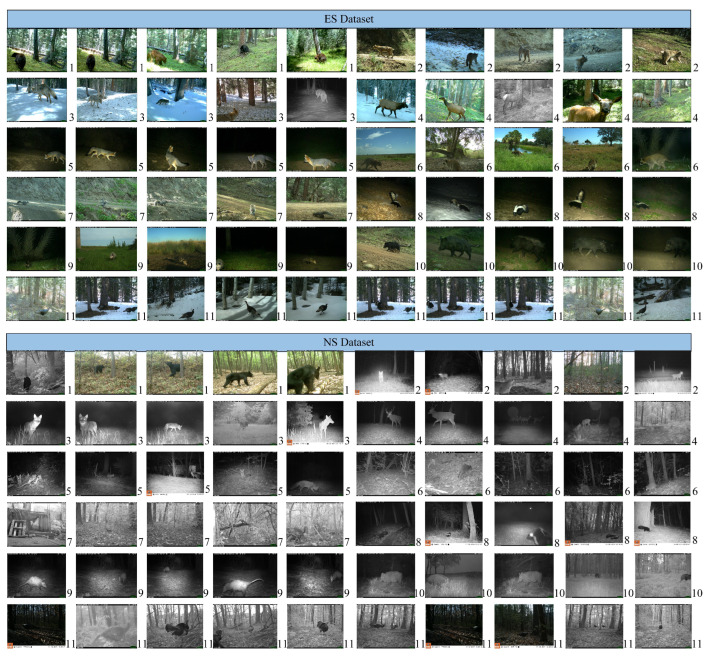
Images of 11 wild animals. Note: 1. bear, 2. bobcat, 3. coyote, 4. deer, 5. fox, 6. raccoon, 7. squirrel, 8. striped skunk, 9. Virginia opossum, 10. wild boar, and 11. wild turkey.

In this paper, the wildlife dataset utilized was derived from two publicly available wildlife datasets, ENA24 [38] and NACTI [39]. These datasets were used to construct two distinct datasets, which are respectively abbreviated as **ES** and **NS** with different distributions but consistent class spaces. They contain 25,591 images of 11 different wildlife species (as shown in Figure 3 and Table 1). These images were captured utilizing infrared camera traps placed in different locations and at different times, resulting in variations in backgrounds, animal poses, and other factors. These distribution differences mainly arise from variations in lighting conditions, camera angles, backgrounds, vegetation, and colors. In this study, each image in the wildlife dataset was resized to a unified size of 224×224 before training the model. The dataset was then divided into source and target domains. Based on the optimization criteria of transfer learning models, two transfer learning wildlife recognition tasks were constructed: **ES**→**NS** and **NS**→**ES**.

### 2.2. Joint Adversarial Learning

In this section, a joint adversarial learning method combining conditional adaptation learning and correlation domain alignment is proposed to promote fine-grained and highly transferable feature generation.

#### 2.2.1. Conditional Adaptation Learning

Deep learning has enjoyed tremendous success in the field of image processing, and one important reason is its ability to confuse domain discriminators and achieve domain alignment by learning new features across domains. Therefore, we first focus on how to learn more transferable features across domains. The Domain-Adversarial Neural Networks (DANN) method [31] can generate domain-transferable feature representations via a minimax game strategy. Specifically, the feature extractor *G* and the label classifier *C* minimize the classification error $L_{CE}$ in the source domain, ensuring that the features generated by the deep network are discriminative. At the same time, the feature extractor *G* maximizes the domain adversarial classification error $L_{CE}$, making the feature distribution domain-transferable. This mechanism establishes a competitive relationship between the feature extractor *G*, the label classifier *C*, and the domain discriminator *D*. During the backpropagation optimization process, the gradient from the domain discriminator *D* to the feature extractor *G* needs to be multiplied by a negative constant. The aforementioned domain-adversarial network provides domain-transferable feature information, which is beneficial for training an effective domain adaptation network model and achieving better unsupervised domain adaptation for image classification. However, dataset bias [40] persists in domain-specific feature and classifier layers, and adversarial adaptation on specific layers alone is insufficient to alleviate dataset bias. Additionally, when domain samples contain complex multimodal structures, domain-adversarial neural network models may not effectively capture the multimodal structure [41] of the samples for fine-grained cross-domain feature distribution alignment. This is known as the mode collapse problem in Generative Adversarial Networks. Incorporating category information predicted by classifiers into adversarial domain adaptation helps address the challenges faced by domain-adversarial neural network models.

For this purpose, a reverse focal loss is applied to the domain discriminator *D* to focus on easily discriminable samples, i.e., samples that are difficult to match. As a result, the objective optimization function for the final domain discriminator *D* and feature extractor *G* in adversarial domain alignment is described by Equation (1).

(1) $$L_{Adv}=-\frac{1}{n_{s}}\sum_{i=1}^{n_{s}}\exp\left(D\left(\phi \left(h_{i}^{s}\right)\right)\right)\log\left(D\left(\phi \left(h_{i}^{s}\right)\right)\right)-\frac{1}{n_{t}}\sum_{j=1}^{n_{t}}\exp\left(1-D\left(\phi \left(h_{j}^{t}\right)\right)\right)\log\left(1-D\left(\phi \left(h_{j}^{t}\right)\right)\right)$$

where exp(D(·)) represents the reverse focal weights for each sample in the source domain, and exp(1−D(·)) denotes the reverse focal weights for each sample in the target domain. The connecting variable h=(f,g) is set, where g represents the results of the label classifier, and f represents the features generated by the feature extractor.

#### 2.2.2. Correlation Domain Alignment

The deep domain transfer model obtained through domain-adversarial networks lacks alignment based on the relevant information of two-domain features. It only captures the second-order statistical information of the unexplored samples and includes irrelevant background information in the learned features. The selected cross-domain feature alignment is irreplaceable due to its second-order statistical characteristics, and it contributes significantly to the final prediction. Therefore, in this paper, we introduce a correlation alignment constraint mechanism that aligns the classifier output features of two-domain samples based on second-order statistical information to promote the accuracy of wildlife recognition. Specifically, the calculation formula for the correlation alignment of features output by the label classifiers for the cross-source and target domain is computed by Equation (2).

(2) $$L_{CA}=\frac{1}{4d^{2}}{∥C_{S}-C_{T}∥}_{F}^{2}$$

where *d* indicates the dimension of the features, ${∥\cdot ∥}_{F}^{2}$ denotes the Frobenius norm of a matrix, and $C_{S}$ and $C_{T}$ indicate the correlation matrices of the source and target domain samples, respectively. Their definitions are as follows:

(3) $$C_{S}=\frac{1}{N_{S}-1}\left(B_{S}^{T}B_{S}-\frac{1}{N_{S}}{\left(1^{T}B_{S}\right)}^{T}\left(1^{T}B_{S}\right)\right)$$

(4) $$C_{T}=\frac{1}{N_{T}-1}\left(B_{T}^{T}B_{T}-\frac{1}{N_{T}}{\left(1^{T}B_{T}\right)}^{T}\left(1^{T}B_{T}\right)\right)$$

where $N_{S}$ and $N_{T}$ denote, respectively, the batch sizes of the source and target domain samples. $B_{S}$ and $B_{T}$ indicate the feature representations output by the label classifier *C*, and $1^{T}$ is a row vector consisting of all ones. The features $B_{S}$ and $B_{T}$ can be gradient computed respectively using the chain rule based on Equations (5) and (6).

(5) $$\frac{\partial L_{CA}}{\partial B_{S}^{ij}}=\frac{1}{d^{2}\left(N_{S}-1\right)}{\left({\left(B_{S}^{T}-\frac{1}{N_{S}}{\left(1^{T}B_{S}\right)}^{T}1^{T}\right)}^{T}\left(C_{S}-C_{T}\right)\right)}^{ij}$$

(6) $$\frac{\partial L_{CA}}{\partial B_{T}^{ij}}=\frac{1}{d^{2}\left(N_{T}-1\right)}{\left({\left(B_{T}^{T}-\frac{1}{N_{T}}{\left(1^{T}B_{T}\right)}^{T}1^{T}\right)}^{T}\left(C_{S}-C_{T}\right)\right)}^{ij}$$

where $B_{S}^{ij}$ represents the *j*-th dimension of the *i*-th source sample, and $B_{T}^{ij}$ indicates the *j*-th dimension of the *i*-th target sample.

### 2.3. Cross-Domain Local and Global Representation Learning

**Figure 4:**
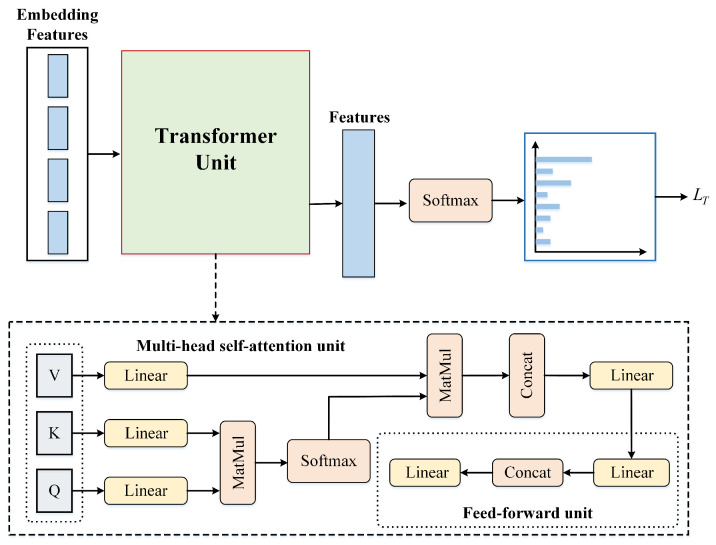
Detailed structure demonstration of the transformer loss.

By utilizing joint adversarial learning as described above, the model can extract the transferable features to reduce the domain discrepancy cross-domain. However, in the hard sample, the decision boundary of the model fails to distinguish them and may mistake these samples. In order to mitigate this issue, cross-domain local and global representation learning is proposed to build the relationship between samples and boost the discriminant ability of the decision boundary. Motivated by the prosperity of transformers in natural language processing and image processing, which aims to build the relationships of the long-range sequence and improve the transferability of features, a transformer transfer loss is designed to construct the relationship of the local and global representations via the transformer unit, as shown in Figure 4.

Specifically, for the local feature $X_{L}$ and global feature $X_{G}$ generated by the feature extractor *G*, we utilize the transformer unit to construct a transformer loss, establishing a relationship with the long-range sequence to enhance the transferability of the features and to facilitate better understanding of the overall structure and relationships within the image. The global feature $X_{G}$ and the local feature $X_{L}$ in each wildlife image can be identified as different patches, which can be termed as token embeddings. All these token embeddings are fed into the transformer unit: the specific operations performed by these embedding features in the transformer unit can be found in Figure 4’s dashed-box section. Then, we can obtain features Z generated based on the local feature $X_{L}$ and global feature $X_{G}$ through the transformer unit. Finally, a transformer loss is defined as follows:

(7) $$L_{T}=-\sum_{i=1}^{C}y_{i}\left(\log\left(\mathrm{Softmax}\left(\left(Z\right)\right)\right)\right)$$

where C represents the number of the class in the wildlife dataset, and $y_{i}$ is the ground-truth label regrading the *i*th class.

### 2.4. Optimization of Deep Joint Adaptation Network

We follow similar steps as [42] to train the source-domain discriminative model C(G(x)) for the wildlife image recognition task via optimizing the parameters of the feature extractor *G* and the label classifier *C*. Therefore, the objective function of optimizing the source domain data is:

(8) $$L_{CE}=\min_{G,C}\frac{1}{n_{s}}\sum_{i=1}^{n_{s}}L_{C}\left(C\left(G\left(x_{i}\right)\right),y_{i}\right)$$

where $L_{C}\left(\cdot \right)$ is a cross-entropy loss function.

In conclusion, by performing joint adversarial learning and cross-domain local and global representation learning in Equations (1), (2), and (7), we can effectively improve the transferability of features and boost the generalization ability so as to learn the benefit decision boundary. Therefore, joining adversarial learning and cross-domain local and global representation learning—that is, the Deep Joint Adaptation Network—the total loss function can be formulated as follows:

(9) $$L=L_{CE}+\alpha *L_{Adv}+\beta *L_{CA}+\gamma *L_{T}$$

where α, β, and γ denote the hyperparameters; these hyperparameters are used to control the degree of influence between different constraints.

## 3. Results

### 3.1. Implementation Details

This section introduces the details of the experiments: (1) We used a 50-layer ResNet [43] as the base network model and conducted experiments on the wildlife dataset. (2) All experiments in this chapter were conducted using the PyTorch framework, and the base network models were pre-trained on the ImageNet dataset. (3) We used mini-batch stochastic gradient descent with a momentum of 0.9. The learning rate strategy described in [42] was employed, where the learning rate $\eta_{p}=\frac{\eta_{0}}{{\left(1+ap\right)}^{b}}$, and *p* linearly varies from 0 to 1 during the training phase. The variable $\eta_{0}$ is the initial learning rate, which starts at zero for the task-specific fully connected layers, and the learning rate for the convolutional layers is 10 times higher than that of the fully connected layers (i.e., the learning rate for the convolutional layers is 0.001, and for the fully connected layers, it is 0.01). The progressive strategy for the domain discriminator is as described in [37], which is $\frac{2}{1+\exp(-\varepsilon p)}-1$ multiplied by α, with α linearly increasing from 0 to 1 and ε set to 10. (4) During the training and testing process, the batch size is set to 128. Additionally, the software and hardware employed in all experiments is reported in Table 2.

### 3.2. Evaluation Metrics

To evaluate the effectiveness of our DJAN model for wildlife image recognition, Accuracy is used as the evaluation metric and is calculated by Equation (10).

(10) $$\mathrm{Accuracy}=\frac{\left|x^{t}:x^{t}\in X^{t}\wedge {\hat{y}}^{t}=y^{t}\right|}{\left|x^{t}:x^{t}\in X^{t}\right|}$$

where $y^{t}$ represents the true class label of the sample $x^{t}$, which is unknown during the learning phase, and ${\hat{y}}^{t}$ represents the predicted class label made by the label classifier for the sample $x^{t}$ in the target domain. Furthermore, this paper also utilizes three evaluation metrics, namely Precision, Recall, and the F1 score, to assess the effectiveness of our method. These evaluation metrics are defined respectively by Equations (11)–(13).

(11) $$\mathrm{Precision}=\frac{\mathrm{TP}}{\mathrm{TP}+\mathrm{FP}}$$

(12) $$\mathrm{Recall}=\frac{\mathrm{TP}}{\mathrm{TP}+\mathrm{FN}}$$

(13) $$F1=\frac{2*\mathrm{Precision}*\mathrm{Recall}}{\mathrm{Precision}+\mathrm{Recall}}$$

where TP (True Positive) indicates the number of correct identifications of a certain wildlife species, FP (False Positive) represents the number of incorrect identifications of a certain wildlife species, and FN (False Negative) is the number of incorrect identifications of a certain wildlife species as another category of wildlife. The F1 score denotes the harmonic mean based on precision and recall.

### 3.3. Comparison with State-of-the-Art Models

In order to reveal the effectiveness of the DJAN approach, we compare DJAN with the state-of-the-art models on the wildlife dataset; these baselines include ResNet50 [43], MMD [44], DANN [31], DCROAL [24], CDAN [37], DSAN [45], BNM [46], HAN [47], and JTN [42]. Table 3 reports the evaluation results on the wildlife dataset with two transfer tasks (i.e., **ES→NS** and **NS→ES**). For fair comparison, the results of the comparative methods are obtained by the authors through experimentation using the source code.

As can be observed from Table 3, the DJAN model overpasses all baselines in both the **ES→NS** and **NS→ES** transfer tasks. This is because DJAN can boost the generalization ability of the network and generate more transferable feature representations via constructing joint adversarial learning and cross-domain local and global representation learning for the wildlife image recognition task. In addition, among the comparison methods, the HAN method [47] achieved the highest transfer task accuracy in wild animal image recognition. It utilizes a conditional domain adaptation and correlation alignment constraint. Compared to the HAN method, our DJAN model achieved a 3.6% (58.2% vs. 54.6%) improvement in average recognition accuracy across two transfer tasks (i.e., **ES→NS** and **NS→ES**). The CDAN method [37] is a further extension of the DANN method [31] that learns cross-domain transferable features by using conditional adversarial domain adaptation. Despite that, our DJAN method still achieves a 7.3% (58.2% vs. 50.9%) higher accuracy than it. Overall, the proposed DJAN model achieved an average accuracy of 58.2% in recognizing 11 categories of wild animals, surpassing all the baseline models. This reflects that our model effectively improves the generalization capacity via employing joint adversarial learning and cross-domain local and global representation learning for wild animal image recognition tasks.

### 3.4. Analysis of Prediction Results from Recognition Models

**Figure 5:**
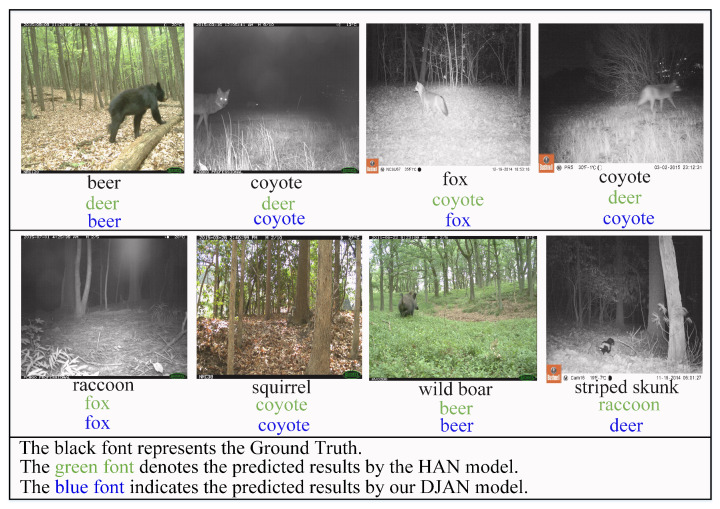
The prediction result comparison of our DJAN and HAN methods on transfer task **ES→NS** for wild animal images.

This section demonstrates that our DJAN model can correctly classify some samples, while the HAN method suffers from misclassification, as shown in Figure 5. The main reason for these misclassifications is that some images were only captured from the back or side of the target animal or the target was in a dark environment where its species-specific image features were not displayed. Therefore, it is difficult for the model to correctly identify them. This once again confirms the effectiveness of our proposed method for improving the transfer performance of the model and consequently enhancing its predictive accuracy by employing conditional adaptation learning, correlation domain alignment, and cross-domain local and global representation learning for wildlife image recognition.

### 3.5. Analysis of Model Performance Using Different Evaluation Criteria

To comprehensively evaluate the performance of our method in wildlife image recognition, we conducted experimental tests using four evaluation metrics: Accuracy, Precision, Recall, and F1 score, on the transfer task **ES→NS**. The experimental results are shown in Table 4. The trends are consistent with the results in Table 3. Our method achieved the highest wildlife image recognition accuracy, precision, recall, and F1 score, with an average accuracy of 48.8%, precision of 0.43, recall of 0.40, and F1 score of 0.35. Comparing to the best results from the comparative methods, our method exhibited improvements of 4.4%, 0.08, 0.05, and 0.03, respectively, further validating the effectiveness of our DJAN approach.

### 3.6. Ablation Study

We conducted ablation experiments to verify the effectiveness of each constraint in our approach on the wildlife dataset, and the results are reported in Table 5. We observed the following: (1) “ResNet50” indicates the results yielded by directly fine-tuning ResNet50 [43] on the wildlife dataset, while “DJAN w/o Adv” denotes the results obtained solely using the deep discriminative feature learning network. Even when only utilizing the correlation alignment adaptation learning and transformer transfer loss, our approach achieves better domain adaptation wildlife image recognition accuracy compared to the ResNet50 method. This suggests that correlation domain alignment and cross-domain local and global representation learning contribute to learning cross-domain transferable features in our approach and achieves higher transfer gains. (2) “DJAN w/o CA” presents the results obtained by removing the correlation domain alignment constraint. Our approach achieves an improvement of nine percentage points (58.2% vs. 49.2%) compared to “DJAN w/o CA”. This improvement is attributed to the ability of the “DJAN w/o CA” to enhance feature transferability. (3) “DJAN w/o T” indicates the results obtained via solely utilizing conditional adaptation learning and the correlation domain alignment constraint. On **ES→NS** and **NS→ES** transfer tasks, our approach outperforms the baselines in achieving the best wildlife image recognition results. (4) Compared to our DJAN approach, the “DJAN method w/o T” model achieves an accuracy below 7.9% (50.3% vs. 58.2%). This further quantitatively validates the effectiveness of cross-domain local and global representation learning, enhancing the accuracy of domain adaptation wildlife image recognition.

### 3.7. Deep Joint Adaptation Network Convergence

**Figure 6:**
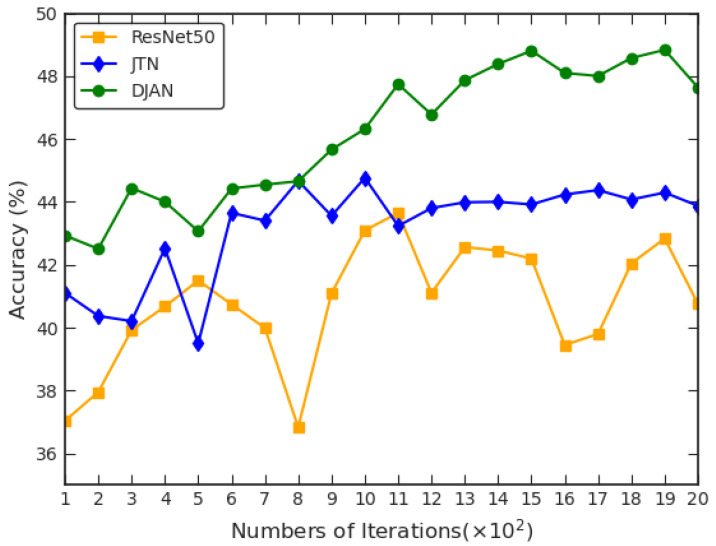
The curve of test accuracy based on ResNet50, JTN, and DJAN on transfer task **ES→NS**.

To investigate the convergence performance of the proposed DJAN model compared to the comparative methods, we conducted the test accuracy experiments on the transfer task **ES→NS**, as shown in Figure 6. By Figure 6, we can observe that the recognition accuracy of our DJAN method gradually increases as the number of iterations increases and then exhibits a stable trend. Moreover, we can also see that although the JTN method achieves stable convergence performance similar to our DJAN model, the convergence performance of our DJAN model is significantly superior to that of the JTN method throughout the entire convergence process. Therefore, the substantial performance improvement for the wildlife image recognition task across domains further reflects the advantages of our DJAN.

### 3.8. Parameter Sensitivity Analysis of the DJAN Model

As shown in Table 6, Table 7 and Table 8, we investigated the sensitivity of hyperparameters α, β, and γ on the transfer tasks **ES→NS** and **NS→ES**. The strategy we adopted was as follows: if we wanted to determine the value of parameter α, we fixed the values of parameters β and γ and obtained the optimal value for parameter α. Similarly, we followed the same process to determine the optimal values for parameters β and γ: by fixing the values of the other parameters. From Table 6, Table 7 and Table 8, we can observe the following: (1) The parameter α changes within the range of [0.2,0.4,0.6,0.8,1.0,1.2]. The transfer performance of our method in this paper initially increases and then decreases. As α increases, the performance first increases and then decreases. Therefore, we set the parameter α to 1.0. (2) The parameter β is optimized within the range of [0.2,0.4,0.6,0.8,1.0,1.2], the accuracy of wildlife image recognition using our method initially stabilizes and then increases before eventually decreasing. Therefore, the value of parameter β is set to 0.25. (3) The parameter γ is optimized within the range of [0.15,0.25,0.35,0.45,0.55,0.65], the transfer gain of the DJAN method first increases steadily and then decreases. Therefore, in our DJAN method, the parameter γ=0.25.

## 4. Discussion

By joining adversarial learning image recognition and vision transformer study research, a Deep Joint Adaptation Network is proposed for a wildlife image recognition model that can both accurately recognition wildlife and enhance generalization ability. Different from domain adaptation wildlife image recognition, such as [20,21,22], the proposed method generates domain-transferable feature representations across domains by the correlation alignment constraint, conditional adversarial training, and cross-domain local and global representation learning method. The correlation alignment constraint and the strategy of conditional adversarial training improve the capability of individual domain adaptation modules. In addition, a transformer unit is utilized to capture the long-range relationships between the local and global feature representations, which facilitates better understanding of the overall structure and relationships within the image. In the study of wildlife image recognition, some deep learning models, such as [10,11,12], also yielded significant recognition results. However, when these methods are confronted with datasets where the feature distribution is significantly affected by factors such as different backgrounds, lighting conditions, and varying scales of capture, the recognition performance can degrade significantly. Moreover, our method avoids the use of costly sample annotation information, which promotes the application of wildlife image recognition in real-world scenarios.

As shown in Table 3, Table 4 and Table 5, it can be observed that conditional adaptation learning and correlation domain alignment obviously further promote the ability of the model to extract more domain-transferable features. This reflects that the combination of the two could further enhance highly transferable feature learning, strengthening the complementarity between adversarial feature learning and domain correlation alignment and thus achieving higher accuracy in wildlife image recognition. However, this mechanism does not consider the long-term relationship between local and global feature representations, which can help to better understand the overall structure and relationships within the image. In this paper, with the help of the domain adaptation strategy of conditional adaptation learning and correlation domain alignment, we designed a transformer loss constraint to capture the long-range relationships between the local and global feature representations, which facilitated better understanding of the overall structure and relationships within the image. With the utilization of the transformer loss to guide the DJAN model learning cross-domain local and global representation, the DJAN model yielded an accuracy of 58.2%, which achieved a 7.9 percentage point improvement in **Accuracy** compared to utilizing conditional adaptation learning and the correlation domain alignment constraint.

The proposed method for wildlife recognition in this paper has indeed improved the recognition accuracy. However, there are still instances where certain individual wildlife species are misclassified (as shown in Figure 5). The main reason for this is the impact of the wildlife photography environment on the transferability of features and the recognition results. Therefore, it is worth considering the introduction of image enhancement algorithms to mitigate the influence of complex background factors and to improve the effectiveness of the model. Furthermore, the next step of this research involves deploying the proposed wildlife recognition model on edge devices to achieve real-time, efficient, and privacy-secure wildlife monitoring at the edge.

## 5. Conclusions

This study presented the Deep Joint Adaptation Network (DJAN), a novel network to address the issue of weak generalization in wildlife image recognition by leveraging the principles of transfer learning. DJAN incorporates two key components: the correlation alignment constraint and conditional adversarial training, which collectively enhance the adaptability of individual domain-adaptation modules. Additionally, transformer units are employed to capture long-range relationships between local and global feature representations, enabling a more comprehensive understanding of the image’s overall structure and relationships. Experimental evaluations conducted on wildlife datasets testify of the effectiveness of the DJAN model, yielding state-of-the-art results. Compared to baseline methods, our DJAN approach achieved an average accuracy improvement of 3.6 percentage points for the classification of eleven wildlife species. These findings highlight the potential of DJAN in advancing wildlife image recognition and its application in the conservation and monitoring of diverse wildlife species in open environments. Further research can explore additional optimizations and extend the use of DJAN to enhance our understanding and preservation of wildlife biodiversity.

## Figures and Tables

**Table 1:** Statistics of the wildlife image dataset.

	bear	bobcat	coyote	deer	fox	raccoon
**ES**	2870	455	1814	4336	1866	1376
**NS**	867	328	181	2357	427	142
	squirrel	striped skunk	virginia opossum	wild boar	wild turkey	
**ES**	1386	1323	110	1893	815	
**NS**	927	295	715	819	289	

**Table 2:** The hardware and software configuration of the experiment.

Test Environment	Type
Operating System	Ubuntu 16.04
Framework	PyTorch 1.4.0
CPU	Intel(R) Core(TM) i9-10900K CPU @ 3.7G Hz
GPU	GeForce RTX 3090Ti (24 G/Nvidia)
RAM	32 G
Programming Language	Python 3.6

**Table 3:** Accuracy performance comparison of the DJAN method and the baselines on wildlife dataset.

Method	ES→NS	NS→ES	Avg.
ResNet50	41.4	55.4	48.4
MMD	44.0	59.2	51.6
DANN	42.8	51.6	47.2
DCORAL	44.1	55.1	49.6
CDAN	45.5	56.2	50.9
DSAN	41.5	53.7	47.6
BNM	28.0	60.1	44.0
HAN	47.2	62.0	54.6
JTN	44.4	62.5	53.5
DJAN	48.8	67.5	58.2

**Table 4:** Performance analysis of wildlife image recognition models under different evaluation criteria.

Methods	Accuracy	Precision	Recall	F1
DCORAL	44.1	0.38	0.29	0.24
JTN	44.4	0.38	0.37	0.32
DJAN	48.8	0.46	0.42	0.35

**Table 5:** Results of ablation studies on wildlife dataset.

Methods	ES→NS	NS→ES	Avg.
ResNet50	41.4	55.4	48.4
DJAN w/o Adv	46.2	53.7	50.0
DJAN w/o CA	40.5	57.9	49.2
DJAN w/o T	43.3	57.3	50.3
DJAN	48.8	67.5	58.2

**Table 6:** The experimental comparison results of different hypermeter α weights on wildlife dataset.

α	0.2	0.4	0.6	0.8	1.0	1.2
**ES→NS**	41.3	43.4	45.8	41.7	48.8	46.4
**NS→ES**	56.9	57.1	56.5	60.2	67.5	58.0

**Table 7:** The experimental comparison results of different hypermeter β weights on wildlife dataset.

β	0.2	0.4	0.6	0.8	1.0	1.2
**ES→NS**	41.3	42.4	43.8	42.7	48.8	42.1
**NS→ES**	54.0	56.8	53.4	54.6	67.5	58.0

**Table 8:** The experimental comparison results of different hypermeter γ weights on wildlife dataset.

γ	0.15	0.25	0.35	0.45	0.55	0.65
**ES→NS**	43.8	48.8	40.8	39.8	42.6	40.4
**NS→ES**	51.5	67.5	55.1	56.1	58.8	55.1

## Data Availability

No data were used for the research described in the article.
